# Lepirudin as an alternative to "heparin allergy" during cardiopulmonary bypass

**DOI:** 10.1186/1749-8090-6-44

**Published:** 2011-04-08

**Authors:** Haralabos Parissis

**Affiliations:** 1 Cardiothoracic Dept, Royal Victoria Hospital, Grosvernor Rd, Belfast, BT12 6BA, Northern Ireland

## Abstract

A treatment strategy of a difficult and unusual problem is presented. We are reporting a case of a patient who had a documented allergy to heparin and required Cardiac surgery for an ASD closure. The anticoagulation regime used during cardiopulmonary bypass was lepirudin based.

This report indicates that r-hirudin provides effective anticoagulation, however unless ECT is monitoring, post operative hemorrhage is encountered. Therefore this case is unique not only because of its rarity but also by the fact that it presents the caveats encountered when ECT is not available.

## Introduction

Traditionally in our effort to maintain optimal cardiopulmonary bypass during cardiac surgery, high dose unfractionated heparin is being used; however there are conditions that the use of heparin is contraindicated. Various thrombin inhibitors could theoretically being used instead, with the favor being Hirudin and lately bivalirudin

### Hirudin

Hirudin is a potent natural direct thrombin inhibitor that is derived from the salivary glands of the medicinal leech, *Hirudo medicinalis *[1]. It is a 65-amino-acid polypeptide that forms a tight, irreversible 1:1 complex with thrombin (1 molecule of hirudin binds with 1 molecule of thrombin).

Hirudin shows both direct anti-Xa activity as well as activation of antithrombin III [2]. It is the most potent and specific thrombin inhibitor known. Uunlike heparin, it is not inactivated by Platelet Factor 4 (PF4), and also can inhibit thrombin bound within the clot [3]. Hirudin is now produced, by using recombinant technology (r-hirudin). Two r-hirudins have been commercially produced (lepirudin and desirudin); however, lepirudin has been more extensively studied and is the focus of this review.

Lepirudin is an anti-thrombotic recombinant DNA form of hirudin derived from yeast cells. Each vial of Refludan contains 50 mg of lepirudin. It is normally used in adult patients requiring anticoagulation who have Heparin Induced Thrombocytopenia (HIT) type II [4].

Two binding sites are present on the thrombin molecule: the active site that catalyzes the majority of the functions of thrombin, and the -brinogen-binding site that mediates binding of thrombin to -brinogen.

Hirudin (lepirudin) binds irreversibly to both the active site and the -brinogen-binding site. Therefore lepirudin is a bivalent direct thrombin inhibitor. The amino-terminal domain binds to the active site of the thrombin molecule and the carboxyterminal domain interacts with the -brinogen-binding site.

The drug distribution follows a two -compartment model with distribution essentially confined to extracellular fluids. There is no known antidote. Clearance from the body is mainly via the kidneys and therefore patients must have normal kidney function if they are to receive this drug.

The most common side effect of the drug used in non-surgical cases is bleeding. The extent of the bleeding following Hirudin administration ranges from mild bruising to severe bleeding (incidence >10%) which can be fatal (incidence 1%). Other rare complications include allergic skin reactions, anaphylactic reactions and injection site pain [5].

We are reporting a rare case of a patient who had an allergy to heparin and required Cardiac surgery for a closure of a large atrial septal defect.

To the best of my knowledge there is only one similar report in the literature that discusses the problem of allergy to heparin in a patient requiring CPB [6]. Moreover this case has a didactic character, because it presents the caveats encountered when ECT is not available.

## Case report

A 30 year old female (47 Kgr) was admitted to the hospital with constitutional symptoms and a large ejection murmur. A large 3 by 4.5 cm secundum Atrial Septal Defect (ASD) was diagnosed using Trans-Thoracic Echocardiogram. There was also a history of transient neurological deficit that was thought to be the result of paradoxical emboli across the ASD.

Further examination revealed a dilated right atrium and moderate pulmonary hypertension. The patient was started on a prophylactic Tinzaparin and subsequently developed generalized itching, flushing, bronchospasm, a widespread urticarial red rash and hypotension. Parenteral H1 antagonists, and epinephrine was administered promptly and the patient was resuscitated with fluids and intravenous steroids, systemically. The symptoms fully resolved in 12 hours. Unfortunately, skin testing is equivocal in diagnosing heparin allergy; Furthermore ELISA screening for Heparin/PF4 antibodies is also not consistent test and "systemic heparin testing" was decided against, due to the previous generalized reaction and the subsequently anticipated dangers involved.

The patient was commenced on warfarin, in view of the history of TIAs. Cardiac angiography revealed normal coronary arteries and confirmed the presence of a large ASD with left to right shunt and was referred to the Cardiac Surgical service for repair of the ASD. Due to the high likelihood of allergy to heparin and in the absence of standardized tests for heparin hypersensitivity, the options available were to either desensitize the patient to heparin or to use an alternative anticoagulant. Following discussions with haematologists and immunologist, it was decided to utilize an alternative anticoagulant during the repair procedure i.e. Lepirudin, trade name Refludan (Schering AG Germany).

During the procedure, a standard non-heparin coated CPB circuit was set up in advance (Terumo Capiox hard-shell venous reservoir and oxygenator). This was primed with as usual with 1200 mls of Hartmann's solution, 50 mmol Sodium Bicarbonate and 25 g mannitol. The 5000 I.U. of heparin normally added to the prime was substituted with 0.5 mg/kg (25 mg) of Lepirudin. Pre-operative aPTT/INR and ACT tests were performed and normal results received (see table 1 below). Pre-op Hb was 12.0 g/dl.

**Table 1:** ACT, APTT and INR values Peri and Post-operatively; Also blood loss, Hb concentration and blood product use peri-operatively, is reported

Time (Hours)	ACT (sec)	APTT (sec)	INR	Blood loss (ml)	Hg (mg/dl)	Blood products
**Operating Theatre**						

10:15 am ( Pre-op)	132	29.4	1.3	140	12.0	

11:00 (post-lepirudin)	>1300	>200	10.0			

11:25 (pre-CPB)	>1500	120	1.9			

11:43 (CPB+5 mins)	>1000	>250	>10			

12:00 (+20 min)	>1000	>250	>10			

12:15 pm (+35 min)	>1000	>250	>10			

12:30 (+50 min)	436	130	6.6			

12:45 ( off CPB)	478	111	3.0			

13:15 (+30 min post-CPB)	476	73	1.9	230	7.0	NONE

**ITU**						

14:10		54	1.5	140	8.9	

15:00		38	1.3	40	7.7	

16:15		47	1.7	30	8.9	RCC:2, FFPs:2

17:00		51	1.5	40	7.5	RCC:2

18:50		40	1.3	3000	6.7	PLTs:2 FFPs:2

20:50		44	1.3	1800	5.9	RCC:6 PLTs:2 CryoP:4 FFPs:2

**Day 1**						

16:00		34	1.2	200	8.9	NONE

18:00		38	1.3	40	9.0	

Note that while the total theatre blood loss was 370 mls, the total ICU blood loss was 5300 mls.

Prior to aortic cannulation, a loading dose of 0.5 mg/kg (25 mg) Lepirudin via the central line was administered. Following a 10 minute interval in order to allow the drug to circulate, the aPTT/INR and ACT tests were repeated. The target values of aPTT >200 sec, INR >2.5 and ACT > 400 sec were achieved before cannulation and initiation of CPB. Repeat testing of both APTT and ACT was performed at 15 minute intervals until CPB was complete. A maintenance infusion dose of 0.15 mg/kg/hour was running during CPB, until 10 minutes before weaning from CPB. The operation involved 40 minutes cross -clamp time and 67 minutes total bypass time. As it is not possible to reverse the activity of Lepirudin, great care was taken to ensure excellent haemostasis throughout the case. Normal coagulation status is only achieved once the Lepirudin has been completely cleared by the kidneys. Immediately following discontinuation of CPB, any Lepirudin infusions running were stopped. The total peri-operatively blood loss was 370 mls. The immediate postoperative Hb was 7.0 g/dl, the drop being mainly attributed to hemodilution with pump prime. The final readings for coagulation tests at completion of CPB were APTT 111 sec, INR 3.0 and ACT 478 sec.

Blood loss increased following patient transfer to the ITU as shown in Table 1. This required aggressive management and the transfusion of multiple blood products over the next 12 hours. These products included 10 units red cell concentrate (RCC), 6 units fresh frozen plasma (FFP), and 2 units of pooled platelets. Four hours post-op on the advise of the consultant hematologist a fibrinogen assay was performed. The result (0.98 g/l) showed the fibrinogen levels to be low (normal range 1.7- 4.1 g/l).

Four units of cryoprecipitate were subsequently transfused. This successfully returned fibrinogen levels to normal as repeat fibrinogen assays, performed 2 hours later, demonstrated (result 2.0 g/l). Blood drainage was markedly reduced following the cryoprecipitate transfusion. Chest drainage in the first 24 hrs post-operatively was in excess of 5 litres. The chest drains were removed and the patient returned to the ward on the 4^th ^post-operative day.

## Discussion

Immune-mediated allergic reactions to heparin have been encountered more often recently, due to the abundance use of this substance for prophylaxis or treatment of Coronary syndromes.

### Types of immune-mediated reactions to heparin

Immediate type I hypersensitivity reaction, (experienced by the patient of the current case report), is a IgE mediated hypersensitivity that can lead to urticarial rash, asthma and anaphylaxis.

Heparin-induced thrombocytopenia type II, is an immune-mediated condition that occurs when predominantly IgG antibodies are produced, against platelet factor 4 (PF4)-heparin complexes. The binding of heparin to PF4 results in the expression of several antigenic sites, which trigger the production of IgG [7].

Subsequently, the large complex of heparin, PF4, and IgG results in platelet activation, platelet destruction, and release of prothrombotic microparticles from platelets and finally disturbance of endothelial cells [8]. The "end result" leads to a paradoxic prothrombotic state (low platelet count, arterial and venous thrombosis). This is what is been considered as the "white clot" syndrome due to platelet-to-platelet adhesions without erythrocytes being trapped in the clot.

It has been reported that HIT II is identified in 1% of cases in patients who undergo open heart surgery [9].

This case was unusual in that it involved a patient with heparin type I allergy. A desensitization protocol has been advocated for patients with heparin allergy, but its efficacy is still anecdotal. Alternative anticoagulation strategies when CPB is necessary have been predominantly suggested in patients with type II reactions. Lepirudin is a potent antithrombin, very effective in inhibiting both free and clot-bound thrombin [10]. There are numerous reports of Lepirudin been used as an alternative anticoagulant to Heparin during CPB in patients with HIT [11,12].

The difficulties with the use of lepirudin for CPB are twofold. First, the suitable laboratory tests for assessing its effect are frequent unavailability in standard hospital laboratories. Furthermore, the unavailability of a reversing agent greatly increases the potential for peri-operative bleeding. These difficulties can be addressed with the provision of adequate tests, (aPTT/INR) or ideally the ecarin clotting time (ECT) test and meticulous peri-operative haemostasis.

### Lepirudin regime for CPB

The use of lepirudin, to systemically anticoagulate a patient during CPB, instead of heparin has important implications. It renders the standard ACT testing, inadequate. Moreover, lepirudin cannot be reversed, as with the heparin-protamine combination, therefore it's use may result in greater peri-operative bleeding.

A low dose of lepirudin is usually recommended for the treatment of HIT and higher doses are required for patients with HIT and established thrombosis or patients requiring CPB procedure [4].

The dosage regime of the drug for CPB used for this case was a 0.5 mg/kg intravenous (IV) bolus with 0.5 mg/kg added to the pump prime and a maintenance dose of 0.15 mg/kg/hr given by infusion. Variations in dosage regimes for Lepirudin use during CPB have been described by different authors [4,12,13]. The most common dosage regime is 0.25-0.4 mg/kg I.V bolus, 0.20 mg/kg in the pump prime and 0.15 mg/kg/hr maintenance dose given by infusion when the monitoring tests fall below acceptable limits [13].

There is no direct correlation between Lepirudin levels and ACT values, however different anticoagulation monitoring methods have been tried where Lepirudin is in use; aPTT should be maintained in excess of 200 sec & the INR should be maintained between 2- 2.5.

The ACT does not test for factor-Xa blockade and therefore there is only a small but incremental change in the ACT with hirudin. The ecarin clotting time (ECT) is the most suitable test for following dosing of lepirudin. Ecarin is derived from the venom of the snake Echis carinatum. Ecarin converts prothrombin to meizothrombin, which has moderate clotting activity. Meizothrombin binds avidly to direct thrombin inhibitors such as lepirudin. Therefore, when all of the lepirudin has been neutralized by meizothrombin in the blood sample, clotting will be initiated. There is a direct relationship between therapeutic levels of lepirudin and prolongation of ECT during CPB. There are however, a great number of questions needing to be answered. Do temperature, hemodilution, severe platelet function abnormalities, the usage of anti platelet agents such as abciximab or extremely low fibrinogen concentrations affect the ECT? Lastly, relatively normal serum levels of pro-thrombin and -brinogen are required for accurate ECT results. Degradation and elimination of lepirudin occur primarily in the kidneys; the lepirudin plasma elimination half- life is approximately 80 minutes in normal subjects, however plasma half-life may be greater than 120 hours in the presence of renal impairment. Therefore lower doses of lepirudin are required under such conditions.

There are no antidote agents for the anticoagulant effect of lepirudin. Koster et al [12] have published on the use of ultra-ltration to enhance the elimination of lepirudin during CPB; they concluded that Plasmapheresis-lter systems were more effective in removing lepirudin than hemo-lter systems (60%-70% v 15%-40% reduction in plasma concentrations of lepirudin, respectively).

Broadly speaking, inadequate anticoagulation is occur at lepirudin levels less than 1.8 to 2.0 μg/mL. In contrary with lepirudin concentrations greater than 4.5 μg/mL the incidence of high postoperative blood loss becomes significant. The recommended target level of lepirudin during CPB, based on various studies [13-15] is 3.5 to 4.5 μg/mL.

The initial short half-life of the drug (10 min) and the leaking to the extravascular space makes estimation of the correct dosage difficult. The post-lepirudin INR in the above case was initially excessive (10.0). It fell rapidly to 1.9 i.e. to within the recommended levels (2.0-2.5) before CPB was commenced. On initiation of CPB the INR again rose, presumably due to the pump-prime drug dose. This pump-prime lepirudin dose (0.5 mg/kg) was the upper limit of that recommended [16]. The resulting high INR results (>10) that were maintained for approximately one hour of CPB may indicate that the pump -prime dose of lepirudin could be reduced in future cases. The maintenance infusion should be adjusted when INR results exceed the target range during CPB. The INR fell to within this recommended range by 30 mins post-CPB and remained low throughout the entire post-op period. The later post-op bleeding may be a "heparin- rebound "(lepirudin rebound in this case) type effect of the extravascular space Lepirudin re-entering the vascular system post-operatively. However one would expect to see a simultaneous increase in the INR if this were the case. This did not occur. The severe post -operative coagulopathy experienced in this case, (also reported by other groups [17,18]), requiring the transfusion of a large number of blood products, highlights the difficulties of using Lepirudin as a systemic anticoagulant for CPB, especially when ECT is not available. The prompt transfusion of appropriate blood products in adequate amounts may also be necessary in the post-operative period. Fibrinogen assaying and cryoprecipitate transfusions should be used where indicated.

Lepirudin might well be recommended to be available in all centers that perform cardiac surgery. Lepirudin, however, is not the ultimate anticoagulant for CPB to replace unfractionated heparin. It requires renal clearance, increases bleeding diathesis and it is also antigenic. It is almost the same size molecule as both protamine and aprotinin. Both of these agents are known for their antigenicity. As per Song et al [19] lepirudin, being a foreign amino acid, will produce its own incidence of allergic reactions.

Bivalirudin could have possibly been alternative therapy especially following increasing evidence on its safety and efficacy [20]. However the current evidence regarding the properties of bivalirudin where not known at the time of the reported case. Retrospectively, taking into account the increased possibility of anaphylaxis after exposure to lepirudin, the unavailability of the ecarin clotting time (ECT) test in some European institutions [21], and the difficulties we faced in terms of monitoring anticoagulation and postoperative excessive bleeding we would consider bivalirudin rather than lepirudin, in a similar case in the future.

In summary, lepirudin provides effective CPB anticoagulation but induces a higher postoperative blood loss than heparin, especially when ECT test is unavailable. Lepirudin should be restricted to patients undergoing CPB who cannot be exposed to heparin. For future such cases we would endeavor to use the ECT test, in conjunction with aPTT/INR testing for optimal monitor blood lepirudin levels peri-operatively. The drug dose, particularly in the pump prime, should be reduced to the lower recommended level of 0.20 mg/kg. The lepirudin infusion should be adjusted to maintain the INR within the recommended limits. The use a cell-saver post-operatively, in order to process residual pump blood and shed chest-drain blood, should also be considered. This would reduce the post-operative lepirudin levels in transfused blood.

Finally, being adequately prepared for future cardiac surgical patients requiring systemic alternative to heparin anticoagulation, is particularly important, as the incidence of HIT in these patients is expected to increase. This maybe due to the increasing number of hospitalized patients in cardiac wards, awaiting surgical intervention, many of whom would be on Heparin therapy with an increased risk of developing HIT.

## Competing interests

The author declares that they have no competing interests.

## Authors' contributions

HP conceived the study and wrote the MS

## Authors statement

All authors read and approved the final manuscript
